# Type 2 diabetes mediated heart failure: focus on early recognition and clinical strategies

**DOI:** 10.3389/fendo.2025.1630686

**Published:** 2025-09-10

**Authors:** Xi Chen, Wei Li, Junwei Zheng, Meinv Huang, Jinxi Wang, Meifang Wu

**Affiliations:** Department of Cardiology, Affiliated Hospital of Putian University, Putian, Fujian, China

**Keywords:** type 2 diabetes mellitus, diabetic cardiomyopathy, heart failure, risk stratification, prevention

## Abstract

Type 2 diabetes mellitus (T2DM) is an important risk factor for the development of heart failure (HF), both directly by impairing cardiac function and indirectly through related conditions such as hypertension, coronary heart disease, renal dysfunction, and other metabolic disorders. The prevention of T2DM-related HF is a comprehensive management process involving complex and multifactorial pathogenic mechanisms. An in-depth exploration of the pathophysiological and clinical risk factors of HF in T2DM can assist clinicians in identifying individuals at high risk of HF, enabling early intervention measures to prevent its onset. In this review, we present data on the pathophysiology and epidemiology of T2DM-mediated HF, clinical phenotypic features of cardiomyopathy, and summarize clinical risk factors predicting HF development identified in multiple studies, risk assessment tools, and clinical trial data on the efficacy of lifestyle modifications, pharmacological treatments, and bariatric surgical interventions. Finally, we discuss best practice recommendations for clinicians, highlight potential limitations and challenges, and propose possible future research directions.

## 1 Introduction

Diabetes mellitus is a chronic metabolic disease influenced by both genetic and environmental factors, with a prevalence that continues to increase annually. The current global prevalence of diabetes among individuals aged 20–79 years is estimated at 10.5% (536.6 million) and is projected to rise to 12.2% (783.2 million) by 2045, with type 2 diabetes mellitus (T2DM), characterized by insulin resistance, accounting for more than 90% of all diabetes cases (1). The coexistence of T2DM with heart failure (HF), either with preserved ejection fraction (HFpEF) or reduced ejection fraction (HFrEF), is common and is associated with a significantly increased risk of HF hospitalization, all-cause mortality, and cardiovascular mortality. The primary factors contributing to HF in T2DM include coronary artery disease, arterial hypertension, and the direct deleterious effects of T2DM on the myocardium (2). Notably, even in T2DM patients with optimal control of traditional risk factors such as glucose, blood pressure, smoking, and hyperlipidemia, the risk of HF remains significant (3). Furthermore, 50–70% of T2DM patients exhibit asymptomatic left ventricular diastolic or systolic dysfunction, which is challenging to detect at early stages due to limitations in diagnostic techniques (4). However, a well-defined framework of assessments and interventions aimed at preventing or delaying HF progression in T2DM is lacking.Recent American Heart Association/American College of Cardiology/Heart Failure Society of America(AHA/ACC/HFSA)Guidelines and the Universal Definition and Classification of Heart Failure categorize the HF process into four stages (5, 6). According to these guidelines, stage A HF is defined as the absence of structural or functional heart disease or abnormal serum biomarkers (5). All individuals with diabetes are considered at higher risk of developing HF and are classified as stage A HF. As myocardial damage progresses, many patients exhibit asymptomatic structural heart disease or elevated filling pressures, defined as stage B HF. This is followed by the onset of HF signs and/or symptoms, representing stage C HF, which ultimately progresses to severe disruption of daily life, classified as stage D HF (7). Data indicate that 5-year survival rates for HF stages are 97% for stage A, 96% for stage B, 75% for stage C, and 20% for stage D (8). Clearly, progression from stage B to stage C HF significantly worsens the prognosis. Therefore, early detection and intervention to delay or prevent the transition from preclinical HF to symptomatic HF are critical public health objectives.

In the effort to prevent the progression of T2DM-related HF, several comprehensive risk assessment tools have been developed to predict HF risk (9–11). The measurement of cardiovascular biomarkers and evaluation of traditional risk factors offer valuable opportunities for risk stratification and individualized HF risk prediction (12).Simultaneously, advancements in imaging techniques have significantly enhanced the sensitivity for detecting early, mild cardiac dysfunction (13, 14).This review explores the pathophysiology, clinical phenotypes, epidemiological features, and risk factors associated with the development of HF in T2DM. It further analyzes the components and efficacy of various risk scoring systems and emphasizes potential preventive strategies. Lastly, based on the currently available, albeit limited, evidence-based medical data, this review proposes both pharmacological and non-pharmacological preventive measures that may effectively reduce the risk of HF progression.

## 2 Pathophysiology of myocardial dysfunction in T2DM

**Figure 1:**
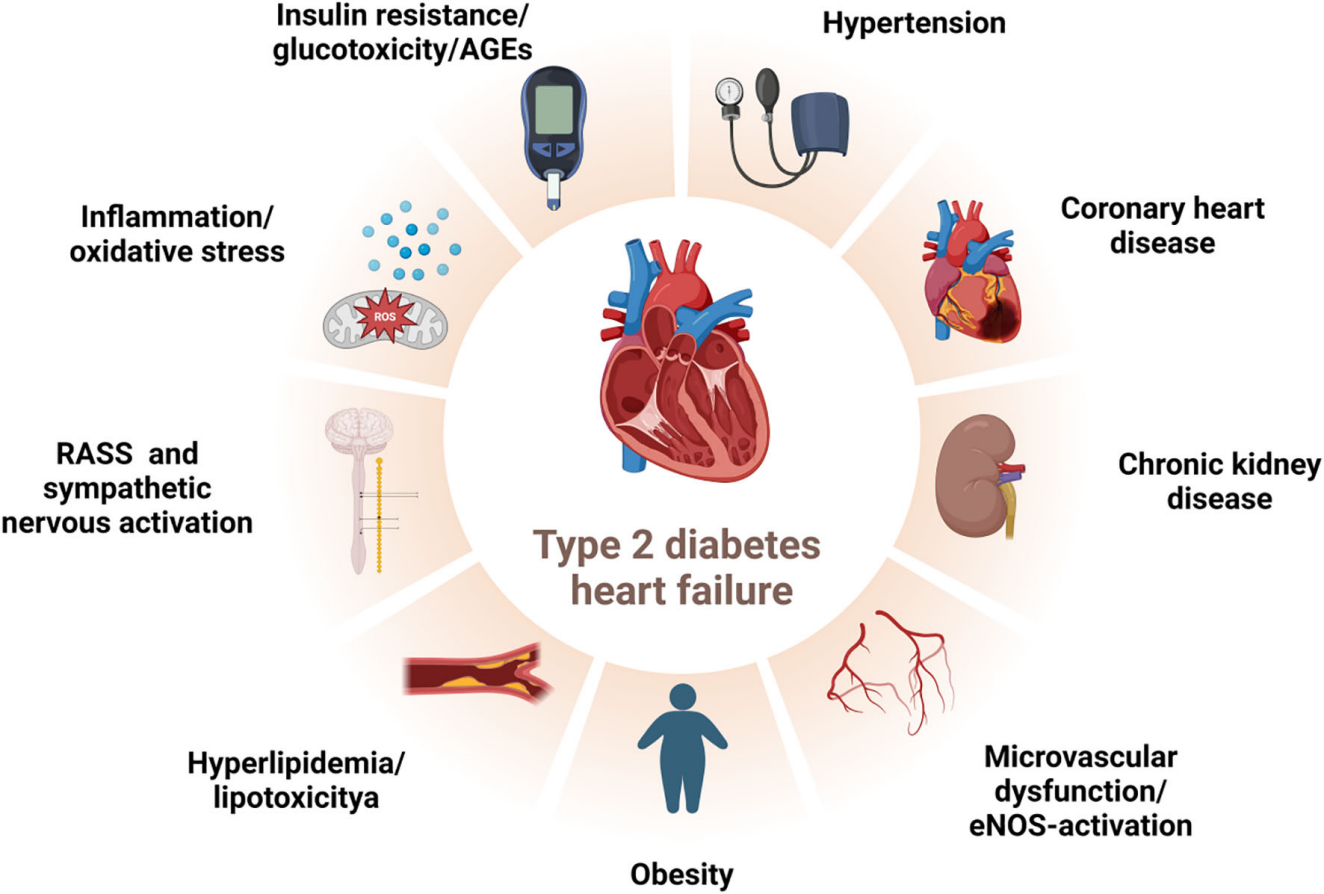
Risk factors for HF in T2DM. The deleterious interplay among direct/indirect hyperglycemic effects, activated neurohumoral abnormalities, and concomitant clinical conditions may reciprocally potentiate each other, forming a vicious feedback loop that culminates in myocardial injury and cardiac dysfunction in T2DM patients. T2DM:type 2 diabetes mellitus, HF: heart failure, RAAS: renin-angiotensin-aldosterone system, AGEs: advanced glycosylation end products, eNOS: endothelial nitric oxide synthase. Created by BioRender.com.

The drivers of myocardial dysfunction in T2DM, in addition to the common coexisting conditions of hypertension and coronary artery disease, include hyperglycemia, insulin resistance/hyperinsulinemia, and impaired glucose tolerance. These factors may exert their effects years or even decades before the clinical onset of T2DM (2, 15). The deleterious effects of these mechanisms are associated with various metabolic abnormalities, such as the deposition of advanced glycosylation end products (AGEs), lipotoxicity, and microvascular dysfunction and rarefaction (16). Glucotoxicity, resulting from hyperglycemia, induces protein glycosylation, leading to an increase in AGEs. These are produced by the non-enzymatic glycosylation of lipids, lipoproteins, and amino acids (17). AGEs alter the mechanical properties of the extracellular matrix by increasing resistance to enzymatic protein hydrolysis in connective tissue and enhancing the cross-linking of collagen and laminin. This, in turn, mediates an increase in myocardial fibrosis, decreased compliance, and left ventricular diastolic dysfunction (18). AGEs can also bind to the receptor for AGEs (RAGE), which promotes the expression of inflammatory genes and increases the production of reactive oxygen species (ROS). This contributes further to inflammation, cardiomyocyte apoptosis, fibrosis, and disturbances in the extracellular matrix, leading to adverse cardiac remodeling and dysfunction (17, 19). Hyperglycemia also exacerbates myocardial pathophysiology through the activation of the renin-angiotensin-aldosterone system (RAAS) and the sympathetic nervous system, as well as coronary microvascular dysfunction secondary to end-glycosylation (20). Insulin resistance, a hallmark of T2DM, is associated with reduced myocardial glucose uptake and compensatory increased uptake of free fatty acids (FFAs) (21). Excessive FFAs in the myocardium can lead to increased lipotoxicity and oxidative stress, resulting in damage to the myocardium (22). Furthermore, normal coronary and myocardial insulin signaling promotes the activation of coronary endothelial nitric oxide synthase (eNOS) and enhances nitric oxide(NO) bioavailability, both of which are critical for optimal coronary microvascular blood flow and myocardial function (17) (**Figure 1**). The harmful interrelationships among these pathophysiological mechanisms may reinforce one another, forming a vicious cycle that mediates myocardial injury and cardiac dysfunction in T2DM (16).

## 3 Phenotypes of T2DM-related cardiomyopathy

In 1972, Rubler et al. reported no evidence of coronary heart disease(CHD) in the autopsy findings of four patients with diabetic glomerulosclerosis and HF. The hearts of these patients exhibited myocardial hypertrophy and fibrosis, suggesting that metabolic factors were responsible for the observed phenomenon (23). Rubler’s observations were later supported by Regan’s study in 1977. This study involved 17 patients with T2DM, in whom CHD had been ruled out by angiography, providing clear evidence of diabetic cardiomyopathy (DCM). The study found elevated left ventricular end-diastolic pressure, reduced left ventricular compliance, and decreased left ventricular ejection fraction (LVEF) with diffuse hypokinesis (24).DCM is characterized by cardiomyocyte hypertrophy, interstitial fibrosis, and impaired coronary microvascular perfusion. In the early stages, DCM usually presents without clinical symptoms, but as the disease progresses, it leads to diastolic or systolic dysfunction. DCM is one of the primary causes of diabetes-related HF and death in patients with T2DM (25). The development of HF in T2DM is not a linear process but involves a series of evolving stages. Long-term exposure to hyperglycemia and insulin resistance (stage A HF) can eventually lead to adverse cardiac remodeling, left ventricular hypertrophy, and cardiac dysfunction (stage B HF) (26). Previous studies have shown that left ventricular diastolic dysfunction can be detected in approximately 75% of T2DM patients, even in the early stages of the disease, including those with normal blood pressure (27). The prevailing view is that left ventricular diastolic dysfunction is one of the first manifestations of DCM and is usually detected earlier than left ventricular systolic dysfunction (28). However, strain analysis and peak systolic velocity measurements have revealed subtle abnormalities in systolic function in 24% of T2DM patients without CHD or left ventricular hypertrophy (29). A recent study showed that altered systolic strain can be detected in patients with T2DM who exhibit normal diastolic function (30). This finding has led to an alternative view that diastolic dysfunction should not be regarded as the first sign of preclinical DCM (14).

Currently, two theoretical perspectives on cardiac dysfunction in DCM are debated: a single clinical phenotype and a dual clinical phenotype (31). The traditional understanding views DCM as progressing from diastolic to systolic dysfunction with structural remodeling, such as left ventricular hypertrophy. The alternative perspective suggests that diastolic and systolic dysfunction in DCM represent a single disease with two distinct phenotypes, which evolve independently into HFpEF or HFrEF (16). Abnormalities in glucose and lipid metabolism, as well as insulin resistance, coexist in obese patients with T2DM, predisposing them to the restrictive/HFpEF phenotype of DCM (diastolic insufficiency).In contrast, autoimmune-associated type 1 diabetes predisposes patients to the dilated/HFrEF phenotype (systolic insufficiency) (16).

## 4 Epidemiology of HF in T2DM

The global prevalence of T2DM has increased by 30% over the past decade, from 333 million people in 2005 to 435 million in 2015 (32). Contemporary data suggest that the overall prevalence of HF in the general population is 11.8%, with a range of 4.7% to 13.3% (33). In numerous clinical trials involving patients with T2DM, the prevalence of HF at baseline ranged from approximately 10% to 30% (34, 35). In the Reykjavik study, the prevalence of HF among individuals with T2DM was 12%, with a higher prevalence in those over 70 years of age—16% for men and 22% for women (34). In the Kaiser Permanente population, the incidence of HF was nearly three times higher in individuals with T2DM under 75 years of age compared to those without T2DM. In the 75–84 age group, the risk of HF was twice as high in those with T2DM as in those without (35). Several factors contribute to these disparities, with gender, race, and other factors potentially influencing the observed differences. Early Framingham studies indicated that men with diabetes were twice as likely to develop HF as non-diabetic men, while women with diabetes were five times more likely to develop HF compared to non-diabetic women (36). Subsequent observational studies have demonstrated gender differences in the association between T2DM and HF risk, with an increased risk in women. Potential explanations for these gender differences include a greater burden of cardiometabolic risk factors, such as elevated body mass index (BMI) and systolic blood pressure at the time of T2DM diagnosis in women, treatment inertia that disproportionately impacts women, and differences in hormonal profiles (37, 38). Similarly, racial differences exist in the risk of HF associated with T2DM. For example, Black individuals with T2DM have a higher risk of developing HF compared to individuals of other races. Racial disparities in HF risk are largely driven by a higher burden of adverse social determinants of health, including lower incomes and limited access to healthcare among Black individuals (39).

## 5 Risk factors for HF in T2DM

The increased risk of HF in T2DM is partly attributable to the direct and indirect effects of hyperglycemia(**Figure 1**). The Atherosclerosis Risk in Communities (ARIC) study provides evidence of subclinical myocardial injury in individuals with prediabetes and T2DM, as assessed by high-sensitivity cardiac troponin T (hs-cTnT) assays. Subclinical myocardial injury increases progressively across the glycemic spectrum, from normoglycemia to prediabetes and diabetes. This is associated with an elevated risk of cardiovascular events, HF, and death, with the highest risk observed in patients with T2DM (40). Blood glucose levels, as measured by glycosylated hemoglobin A1c (HbA1c), serve as an independent biomarker of HF risk in patients with T2DM and even prediabetes (41–43). In T2DM patients, each 1% increase in HbA1c is associated with an 8% higher risk of HF hospitalization or HF-related death (41). In non-diabetic individuals, a 1% increase in HbA1c is associated with a 39% increased risk of HF, independent of other risk factors (43). Additionally, the duration of diabetes is an independent risk factor for HF, with each 5-year increase in the duration of diabetes corresponding to a 17% higher risk of HF (44).

Patients with T2DM often have additional comorbid risk factors for HF, which contribute to indirect myocardial injury. More than 70% of patients with T2DM have elevated blood pressure, and the coexistence of hypertension and T2DM exacerbates vascular remodeling, atherosclerosis, cardiac structural and functional abnormalities, and coronary microvascular dysfunction, all of which increase the risk of HF (45–47). Approximately 60% of individuals with T2DM also suffer from obesity, a common risk factor for both T2DM and HF (48, 49). An earlier study demonstrated that metabolic syndrome (defined by a BMI greater than 29.4 kg/m²) increased the risk of HF more than threefold over 20 years of follow-up. This increased risk persisted even after adjusting for established risk factors for HF (50). Subsequent studies have further shown that central abdominal obesity and high-fat mass are strongly associated with an elevated risk of HF in T2DM patients (51, 52). Chronic kidney disease (CKD) affects approximately 40% of T2DM patients (53).Impaired renal function and albuminuria are important independent risk factors for the development of HF in individuals with T2DM (11, 54). A recent study demonstrated that the degree of elevation in the urinary albumin-creatinine ratio (uACR) was associated with a progressively higher risk of new-onset HF in T2DM. This ranged from microalbuminuria (adjusted hazard ratio [HR], 2.21; 95% CI, 1.59–3.06) to macroalbuminuria (adjusted HR, 6.02; 95% CI, 4.11–8.80) (54). Conversely, slowing the progression of CKD in T2DM patients has been shown to reduce cardiovascular morbidity, particularly HF morbidity and mortality (55). The risk of CHD in individuals with T2DM is at least twice as high as in non-diabetic individuals. This is typically manifested as diffuse, multivessel coronary artery disease or, in some cases, as asymptomatic myocardial infarction, which is a significant cause of cardiac dysfunction, especially HFrEF (56–59). Notably, although some risk factors for HFpEF and HFrEF overlap (e.g., older age, diabetes mellitus, and a history of valvular disease), HFpEF is more commonly associated with women, obesity, and physical inactivity, whereas HFrEF is more commonly associated with men, smoking, and CHD (59–61).

## 6 Risk prediction of HF in T2DM

### 6.1 Clinical risk scores to predict HF risk in T2DM

The importance of risk assessment in cardiovascular disease prevention is increasingly emphasized within the academic community. As a result, several HF risk scores based on clinical risk factors in patients with T2DM have been developed (10, 11, 62–69) (**Table 1**). Among these, two relatively concise and widely validated risk scores are the WATCH-DM(Weight, Age, hyperTension, Creatinine, High-density lipoprotein cholesterol[HDL-c], Diabetes control, Myocardial infarction or coronary artery bypass grafting) risk score and the Thrombolysis in Myocardial Infarction Risk Score for HF in Diabetes (TRS-HFDM).The WATCH-DM risk score is a machine-learning-based methodology designed to predict the 5-year risk of HF in patients with T2DM. The score includes the following variables:BMI, age, hypertension, creatinine levels, HDL-c, fasting glucose, HbA1c, QRS duration, and a history of myocardial infarction or coronary artery bypass grafting (10).The TRS-HFDM score was developed based on results from the SAVOR-TIMI 53 trial and validated in the DECLARE-TIMI 58 trial for predicting the risk of hospitalization for HF. This scoring system, which ranges from 0 to 7, incorporates five independent risk factors: history of previous HF, history of atrial fibrillation, history of CHD, estimated glomerular filtration rate (eGFR), and uACR (11). Both the WATCH-DM and TRS-HFDM scores have been validated in multiple external cohorts, demonstrating adequate identification and risk stratification efficacy (69–72). However, there are some limitations to these risk scores. First, both scores rely solely on clinical risk factors and do not account for the contribution of cardiac biomarkers, such as hs-cTnT and natriuretic peptide concentrations, or some novel biomarkers, in their assessments. This is a significant consideration, as elevated hs-cTnT and natriuretic peptide concentrations are among the strongest predictors of HF in adults, and certain novel biomarkers have prognostic predictive value (73–76). Secondly, dynamic changes in these variables, including indicators related to renal function, are not included in the models. Lastly, the accuracy of these scores in predicting HF risk is limited by the presence of competing risks (77).

**Table 1:** Prediction model of primary HF in T2DM patients.

Author and year of publication	population	Time horizon	Clinical variables	Number of variables(n)	Model presentation	Derivation cohort	Discrimination
Yang et al(2008) (62)	T2DM without HF(n=7,067)	5-year risk	age,BMI,uACR, HbA1c,hemoglobin,CHD	6	Regression coefficients	Electronic medical records	0.85
Pfister et al. (2013) (63)	T2DM without HF(n=4,951)	follow-up 34.5 ± 2.3months	age,renal dysfunction, diuretic use, HbA1c, diabetes duration, LDL-c, heart rate, left bundle branch block, right bundle branch block,microalbuminuria, previous myocardial infarction, pioglitazone treatment	12	Integer score	PROactive	0.75
Hippisley-Cox and Coupland (2015) (64)	T2DM without HF(derivation cohortn=25,480 validation cohort n=8,189)	10-year risk	age, BMI, SBP, cholesterol/HDL-c ratio, HbA1c, deprivation, ethnicity, smoking, diabetes type, diabetes duration, atrial fibrillation, cardiovascular disease, CKD	13	Online calculator	Electronic medical records	0.77
Halon et al. (2017) (65)	T2DM without HF(n=735)	follow-up 8.4 ± 0.6 years	left atrium-to right atrium volume ratio>1,Microvascular disease, SBP	3	Regression coefficients	Cohort study	0.79
Segar et al. (2019) (10)	T2DM without HF(derivation cohortn=8,756 validation cohort n=10,819)	5-year risk	age, BMI, SBP, DBP, QRS duration, myocardial infarction history, coronary artery bypass graft surgery history, fasting plasma glucos, serum creatinine,HDL-c	10	Online calculator and integer score	ACCORD	Training: 0·74 and test: 0·77
Williams et al. (2020) (66)	T2DM without HF(n=54,452)	1-year, 3-year, and 5-year risk	age, CHD, blood urea nitrogen, atrial fibrillation, HbA1c, blood albumin, SBP, CKD, and smoking history	9	Integer score	Electronic medical records	0.78
Pandey et al. (2021) (67)	T2DM and pre-diabetes without HF(n=6,799)	5-year risk	hs-cTnT, NT-proBNP, high-sensitivity C-reactive protein, left ventricular hypertrophy by electrocardiography	4	Integer score	ARIC, DHS, MESA	0.74
Patel et al. (2022) (68)	T2DM without HF (derivation cohort n=5,080 validation cohort *n*=9,155)	12-year risk	NT-proBNP, waist circumference, uACR, eGFR, HbA1c	5	Integer score	Look AHEAD	0.79
Segar et al. (2022) (69)	T2DM and pre-diabetes without HF(n=8,938)	5-year risk	NT-proBNP, age, BMI, SBP, DBP, QRS duration, myocardial infarction history, coronary artery bypass graft surgery history, fasting plasma glucose, serum creatinine, HDL-c	11	integer score	ARIC, CHS, FHS, MESA	0.76
Tao et al. (2023) (54)	T2DM without HF(n=9,287)	median follow-up of 4.05 years	uACR, age, sex	3	Regression coefficient	Electronic medical records	0.78
			uACR, age, BMI, SBP, DBP, FPG, serum creatinine, HDL-c, CHD	9			0.80
			uACR, age, SBP, CHD, atrial fibrillation, HbA1c, albumin, BUN, eGFR, smoking	10			0.81

T2DM:type 2 diabetes mellitus, HF: heart failure, BMI: body mass index, uACR: urinary albumin-creatinine ratio, HbA1c:glycosylated hemoglobin A1c,CHD:coronary heart disease, LDL-c: low-density lipoprotein cholesterol, HDL-c: high-density lipoprotein cholesterol, SBP: systolic blood pressure, DBP: diastolic blood pressure, CKD: chronic kidney disease, hs-cTnT: highly sensitive-cardiac troponin T, NT-proBNP: N-terminal pro B-type natriuretic peptide, FPG: fasting plasma glucose, eGFR: estimated glomerular filtration rate, BUN: blood urea nitrogen.

### 6.2 Value of cardiac biomarkers in predicting HF risk in T2DM

In recent years, cardiac biomarkers have been explored in various ways for the prediction and diagnosis of cardiovascular disease. These include both biomarkers already in clinical use and newly developed biomarkers still under clinical investigation (78). Numerous studies have confirmed the utility of cardiac biomarkers in guiding the prevention of HF in patients with T2DM (67, 68, 75, 79, 80). The traditional biomarker B-type natriuretic peptide (BNP) is secreted from cardiomyocytes in response to myocardial wall stretch, regulated primarily at the transcriptional level. It is released as a precursor protein (pro-BNP), which is subsequently cleaved into biologically active BNP and the inactive peptide N-terminal pro-BNP (NT-proBNP) (81, 82). In the STOP-HF trial, participants older than 40 years of age and assessed to be at high risk of developing heart failure based on traditional risk factors, including T2DM, were randomly assigned to either BNP screening or usual primary care (79). In the BNP screening group, participants with BNP ≥50pg/mL (indicative of stage B HF) underwent echocardiography and received collaborative care between their primary care physician and a cardiologist. This approach resulted in a 45% reduction in the likelihood of progressing to stage C HF and left ventricular dysfunction compared to the usual primary care group.In the PONTIAC trial, patients with T2DM at high risk for HF and NT-proBNP levels ≥ 125 pg/mL benefitted from intensified neurohormonal blockade therapy and cardiovascular disease specialist care, reducing the risk of subsequent HF by 65% (83). Highly sensitive cardiac troponin (hs-cTn) has also been shown to predict the occurrence of new-onset HF in asymptomatic patients and can identify those at high risk for developing HF (80). The American Diabetes Association (ADA) published a consensus report recommending annual measurement of natriuretic peptides or hs-cTn to identify T2DM patients who may be at risk for stage B HF. Thresholds for identifying stage B HF include BNP ≥ 50 pg/mL, NT-proBNP ≥ 125 pg/mL, and hs-cTn ≥ 99th percentile of the upper reference limit of the assay (84).Several studies have evaluated the combination of biomarkers with risk scoring models or the use of multiple biomarkers to predict HF risk in patients with T2DM (67, 69). An analysis of adults with T2DM or prediabetes, without HF at baseline, in four cohort studies (Atherosclerosis Risk in Communities[ARIC], Cardiovascular Health Study[CHS], Framingham Offspring Study[FHS], and Multi-Ethnic Study of Atherosclerosis[MESA])showed that NT-proBNP alone was more effective in predicting HF in subjects with low/intermediate WATCH-DM scores (<13) than in those with high WATCH-DM scores (≥13) (C-index 0.71 [95% CI 0.68–0.74] vs. 0.64 [95% CI 0.61–0.66]). HF risk identification improved when NT-proBNP levels were combined with WATCH-DM scores, showing greater improvement in low/intermediate risk patients [WATCH-DM score <13] than in those at high risk [WATCH-DM score ≥13] (C-index 0.73 [95% CI 0.71–0.75] vs. 0.71 [95% CI 0.68–0.74]) (69). A 2021 multi-cohort analysis developed a four-item scoring system based on three biomarkers—hs-cTnT (≥ 6 ng/L), NT-proBNP(≥125pg/mL),and high-sensitivity C-reactive protein (≥3mg/L)—as well as electrocardiographic left ventricular hypertrophy. This model was used to predict the 5-year risk of HF in T2DM and prediabetes patients with no history of cardiovascular disease. The results indicated that the risk score was well-calibrated and discriminatory in this population (C-index 0.74, 95% CI 0.68–0.80) (67). Furthermore, soluble suppression of tumorigenicity-2 (sST2), a promising new biomarker in the field of HF, has been demonstrated to correlate with early myocardial fibrosis and predict adverse cardiovascular events in T2DM (85). However, its utility in identifying HF risk among patients with T2DM remains to be established.

### 6.3 Assessing HF risk by cardiac imaging techniques

In patients with T2DM, subclinical cardiac damage is a frequent concomitant condition, and echocardiography plays a pivotal role in identifying structural or functional cardiac abnormalities that may contribute to the development of HF (86–88). Three echocardiographic index abnormalities have been proposed as potential diagnostic criteria for DCM: left ventricular hypertrophy, left atrial enlargement, and the presence of diastolic dysfunction. The following specific definitions have been used to assess the prognostic impact of DCM and the subsequent development of HF: least restrictive (at least one atypical echocardiographic abnormality), intermediate restrictive (at least two atypical echocardiographic abnormalities), and most restrictive (at least two atypical echocardiographic abnormalities with elevated natriuretic peptide concentrations). The prevalence of DCM was found to range from 67% (using the least restrictive definition) to 12% (using the most restrictive definition). Regardless of the criteria used to define DCM, diabetic patients with DCM had a significantly higher risk (2–4 times higher) of developing HF compared to patients with diabetes alone (89). Given that diabetic myocardial damage often develops insidiously, the advent of two-dimensional speckle-tracking echocardiography has significantly improved the early recognition of left ventricular dysfunction in DCM in recent years (90, 91). Using the more sensitive left ventricular global longitudinal strain (GLS) technique, early minor abnormalities in cardiac systolic function can be detected (30). Data suggest that approximately 45% of patients with T2DM have subclinical left ventricular systolic dysfunction, and that impairment of left ventricular GLS is associated with subsequent HF (87, 92). A combined approach utilizing echocardiographic features and cardiac biomarker concentrations, which considers the presence or absence of abnormal myocardial structure (e.g., left ventricular hypertrophy or concentric remodeling), function (e.g., diastolic dysfunction or abnormal left ventricular strain), and biomarker concentrations (indicating myocardial stretching or injury), further classifies DCM and stage B HF into four subcategories (Stage B1: Elevated biomarkers with normal cardiac structure and function; Stage B2: Elevated biomarkers with atypical cardiac structural abnormalities but normal cardiac function; Stage B3: Elevated biomarkers with atypical cardiac structural and functional abnormalities; Stage B4: Elevated biomarkers, low ejection fraction, and moderate-to-severe valvular disease). This new classification suggests that the risk of developing stage C HF progressively increases from stage B1 to stage B4 (7). Additionally, the ability of other imaging methods, such as cardiac magnetic resonance and radionuclide imaging, to predict the risk of HF in diabetic patients has not been well studied (93). These cardiac imaging methods are not recommended for routine examination in asymptomatic patients.

## 7 Prevention of HF in T2DM

### 7.1 Lifestyle interventions

A meta-analysis indicated that structured aerobic, resistance, or combined exercise training, along with dietary advice, improved glycemic control and significantly reduced HbA1c in diabetic patients (94). Exercise was associated with enhanced insulin sensitivity, even when weight loss was modest (defined as ≥3% to <5% weight loss) (95). This finding suggests that the relationship between exercise and improved glycemic control may be independent of weight loss. However, the cardiac benefits of exercise in patients with T2DM may be attenuated (96, 97). In a study utilizing tissue Doppler imaging, 176 patients with T2DM were randomly assigned to exercise training or usual care. No significant differences in myocardial strain or tissue velocities were observed between the two groups after 1 year of follow-up (96). In the multicenter Look Action for Health in Diabetes(Look AHEAD)study, although the intensive lifestyle intervention resulted in modest weight loss and a significant improvement in HbA1c, it did not lead to a reduction in the primary composite outcome, which included hospitalization for cardiovascular causes, non-fatal myocardial infarction, non-fatal stroke, or angina (98). Notably, further analysis of the Look AHEAD data revealed that participants who achieved improvements in fitness and weight loss had a lower subsequent risk of HF at the 4 year follow-up.In adults with T2DM, lifestyle interventions were associated with significant reductions in fat mass and lean mass. Reductions in fat mass and waist circumference, but not lean mass, were significantly associated with a reduced risk of HF. Additionally, reductions in waist circumference were significantly associated with a reduced risk of HFpEF, but not with HFrEF (99). Therefore, exercise interventions are more likely to benefit individuals with T2DM and obesity, particularly through effective body fat reduction, reversal of central obesity, and improvements in cardiorespiratory fitness.

### 7.2 Pharmacological interventions

#### 7.2.1 Traditional hypoglycemic agents

Metformin and sulfonylureas are commonly used to achieve glycemic control in diabetic patients. In addition to lowering blood glucose, metformin has been shown to have beneficial effects by stimulating insulin action, reducing inflammation, and improving myocardial energy metabolism (100–102). Despite these multiple mechanisms of cardiovascular benefit, current evidence does not support metformin’s ability to reduce HF in T2DM. A study examining the cardiovascular outcomes of the Diabetes Prevention Program (DPP) Trial and the DPP Outcomes Study (DPPOS) found that metformin intervention failed to reduce the risk of HF in T2DM patients during a median follow-up of up to 21 years (103). Other studies have similarly shown that metformin treatment does not reduce the risk of developing HF (104, 105). Sulfonylureas are associated with an increased risk of adverse events in T2DM patients and a higher incidence of hypoglycemia (106–108). A recent meta-regression analysis of 18 studies evaluating the risk of cardiovascular events associated with sulfonylureas found that treatment with sulfonylureas was linked to an increased risk of cardiovascular death and events (109). In the CAROLINA trial, patients with T2DM were randomly assigned to treatment with either the sulfonylurea glimepiride or the dipeptidyl peptidase 4(DPP-4) inhibitor linagliptin. The rate of hospitalizations for HF was similar in both groups (3.1% for glimepiride and 3.7% for linagliptin) (110). Thiazolidinediones, known for their glucose-lowering effects, are also associated with fluid retention, which increases the risk of HF (111). In the PROACTIVE study, patients with T2DM and a history of macrovascular disease were randomized to receive either pioglitazone or placebo. Pioglitazone was found to increase the rate of hospitalization for HF, although this was accompanied by a reduction in cardiac ischemic events (63). In the RECORD trial, a multicenter, open-label study of T2DM patients, rosiglitazone was associated with more than a two-fold increase in the risk of HF (112). Insulin is often added to the treatment regimen when oral antidiabetic medications fail to achieve adequate glycemic control or when oral agents cannot be used. Mechanistically, insulin can contribute to fluid retention and weight gain, and it has been shown that insulin therapy in T2DM patients is associated with an increased prevalence of HF and cardiovascular disease mortality (113). However, contradictory findings exist. In the ORIGIN trial, which enrolled 12,537 patients with diabetes mellitus or prediabetes and followed them for 6 years, the results showed no increase in the risk of HF in patients treated with insulin glargine compared to those receiving standard therapy (114). Similarly, there was no difference in the incidence of HF between the two groups of T2DM patients randomized to ultra-long-acting insulin degludec and insulin glargine in the DEVOTE trial (115). In summary, although traditional oral hypoglycemic agents and injectable insulin are widely used, most do not have a preventative effect on HF, and some may even be harmful. Therefore, new oral hypoglycemic agents offer therapeutic hope for patients with T2DM at high risk for HF.

#### 7.2.2 Novel hypoglycemic agents

##### 7.2.2.1 Sodium-glucose cotransporter 2 inhibitors

SGLT2 inhibitors block glucose reabsorption in the proximal tubules of the kidney, thereby increasing glucose excretion in the urine and improving glycemic control (116). The three SGLT2 inhibitors approved for clinical use are empagliflozin, dapagliflozin, and canagliflozin (117). Recently, a large body of basic and clinical research has elucidated that the cardioprotective effects of SGLT2 inhibitors in patients with T2DM and non-diabetic patients are independent of their antihyperglycemic effects (118). The EMPA-REG OUTCOME trial evaluated the non-inferiority of empagliflozin compared to placebo for major cardiovascular adverse event (MACE) in 7,020 patients with T2DM at very high cardiovascular risk. The results demonstrated that empagliflozin was superior to placebo in reducing the risk of MACE, as well as decreasing the relative risk of hospitalization for HF by 35% (119). Subsequently, the DECLARE-TIMI 58 trial assessed the cardiovascular safety and efficacy of dapagliflozin in patients with T2DM at relatively low cardiovascular risk. Although dapagliflozin did not significantly alter MACE, it was associated with a significant 17% reduction in the composite endpoint of cardiovascular death or first hospitalization for HF, primarily driven by a 27% reduction in first hospitalization for HF (120). Similarly, the CANVAS trial and the VERTIS CV trial showed a 33% and 30% reduction in the risk of hospitalization for HF, respectively, with the use of canagliflozin and ertugliflozin (121, 122). Moreover, SGLT2 inhibitors have demonstrated significant benefits in reducing the risk of major adverse clinical events in patients with CKD. In patients with T2DM and CKD, canagliflozin reduced the relative risk of renal composite adverse events (such as end-stage renal disease, doubling of serum creatinine, or death from renal causes) by 34%, cardiovascular death, myocardial infarction, or stroke by 20%, and, notably, the risk of hospitalization for HF by 39% (123). The DAPA-CKD study, which enrolled adults with an eGFR of 25–75 mL/min/1.73 m² and a uACR of 200–5000 mg/g, demonstrated that dapagliflozin significantly reduced the relative risk of a composite adverse renal outcome, hospitalization for HF, or cardiovascular death, regardless of T2DM status (124). These findings strongly suggest that SGLT2 inhibitors are effective as therapeutic agents in the treatment of T2DM, cardiorenal diseases, and the prevention of HF. Studies in animal models have also revealed some of the mechanisms by which SGLT2 inhibitors improve cardiovascular outcomes, further supporting their clinical benefits. Empagliflozin has been shown to ameliorate left ventricular diastolic dysfunction in db/db mice fed a high-fat western diet by reducing spontaneous diastolic sarcoplasmic reticulum calcium release (125). In the ob/ob-/- mouse model, empagliflozin induced a shift to a more catabolic state, including lower blood cholesterol and HbA1c, higher glucagon/insulin ratios, elevated ketone levels, and an increase in the L-arginine/asymmetric dimethyl arginine ratio (an indicator of endothelial function). These changes led to improvements in cardiac contractility and coronary microvascular function (126). Additionally, in a non-diabetic model, dapagliflozin alleviated myocardial hypertrophy, fibrosis, and excessive collagen synthesis, resulting in a significant improvement in left ventricular GLS (127). A variety of mechanisms underlying the cardiac benefits of SGLT2 inhibitors have been proposed, including diuresis, reduction of inflammation and oxidative stress, improved cardiac energy metabolism, and better intracellular calcium homeostasis. However, more precise mechanisms need to be further elucidated (128–131).

##### 7.2.2.2 Glucagon-like peptide-1 receptor agonists

GLP-1 receptor agonists bind to GLP-1 receptors on pancreatic β-cells, reducing blood glucose levels by promoting insulin synthesis and secretion, inhibiting glucagon secretion, enhancing glucose utilization by peripheral tissues, decreasing hepatic glucose output, and increasing insulin sensitivity to glucose (132). Studies on the primary prevention of HF in patients with T2DM using GLP-1 receptor agonists have yielded mixed results.The LEADER study, an international multicenter, randomized, double-blind, placebo-controlled trial involving 9,340 patients with T2DM at high cardiovascular risk (81.3% of whom had a history of cardiovascular disease), compared the long-term effects of liraglutide (1.2 mg and 1.8 mg) to those of placebo over a mean follow-up of 3.8 years. This study demonstrated that the composite cardiovascular endpoint (cardiovascular death, nonfatal myocardial infarction, or nonfatal stroke) and HF hospitalization were significantly lower in the liraglutide group compared to the placebo group (133). Subsequent cardiovascular outcome studies involving albiglutide and efpeglenatide in T2DM patients reported a 29% and 39% reduction in the risk of hospitalization for HF, respectively (134, 135). In a meta-analysis of trials evaluating cardiovascular outcomes with different GLP-1 receptor agonists, participants assigned to GLP-1 receptor agonists had a significant 11% lower risk of hospitalization for HF (136). These findings suggest a potential role for GLP-1 receptor agonists in the prevention of HF in patients with T2DM. However, two additional cardiovascular outcome studies involving liraglutide and semaglutide, respectively, demonstrated significant reductions in the risk of MACE, but did not show any effect on the risk of hospitalization for HF (133, 137). Taken together, these results raise questions about the role of GLP-1 receptor agonists in the prevention of DCM. The differences in outcomes may be attributed to variations in patient characteristics, such as obesity, with obese patients being more likely to benefit. These findings highlight the need for further high-quality randomized controlled trials to better understand the potential of GLP-1 receptor agonists in preventing HF in diverse populations of T2DM patients (138).

##### 7.2.2.3 Dipeptidyl peptidase 4 inhibitors

Intestinal insulin-based therapy has emerged as a new strategy for diabetes management, and DPP-4 plays a key role in the clearance of GLP-1 (139). DPP-4 inhibitors improve insulin sensitivity and glucose control by increasing insulin secretion from pancreatic β-cells (140). The SAVOR-TIMI 53 trial enrolled 16,492 patients with T2DM who had a history of, or were at risk for, cardiovascular events. These participants were randomly assigned to receive either saxagliptin or placebo. The results showed that saxagliptin did not affect the risk of MACE but was associated with a 27% increased risk of hospitalization for HF (141). Similarly, a *post hoc* analysis of the EXAMINE trial revealed that alogliptin was associated with a 76% increased risk of hospitalization for HF in patients with T2DM who did not have a history of HF (142). However, the Trial Evaluating Cardiovascular Outcomes With Sitagliptin(TECOS), a randomized, double-blind study enrolling 14,671 patients with T2DM and cardiovascular disease, found that sitagliptin did not increase the risk of hospitalization for HF (143). In two additional cardiovascular outcome trials involving linagliptin, no increased risk of hospitalization for HF was observed (133, 144). In conclusion, these studies suggest that DPP-4 inhibitors may increase the risk of HF in some patients with T2DM. However, they also indicate that certain DPP-4 inhibitors may be safe. Given that other antidiabetic medications are available with favorable safety and efficacy profiles for the prevention of HF, DPP-4 inhibitors should be avoided in patients at risk for developing HF.

##### 7.2.2.4 Renin-angiotensin system inhibitors

Approximately two-thirds of individuals with T2DM also have arterial hypertension (145). For blood pressure management in T2DM, the ADA recommends a target of less than 140/90 mmHg if the estimated 10-year risk of atherosclerotic cardiovascular disease (ASCVD) events is less than 15%. If the risk is 15% or more, the target should be less than 130/80 mmHg (117). Notably, intensive blood pressure lowering (systolic blood pressure less than 120 mmHg) did not provide additional benefits in reducing the risk of HF compared to a target of less than 140/90 mmHg (146). Angiotensin-converting enzyme inhibitors (ACEIs) or angiotensin receptor blockers (ARBs) are the preferred drugs for treating hypertension in T2DM, particularly in the presence of proteinuria (147). In many cases, however, patients treated with optimal doses of ACEIs or ARBs may experience aldosterone escape, leading to activation of the mineralocorticoid receptor(MR) signaling pathway. This can contribute to the development of diabetes-induced HF by promoting fibrosis and insulin resistance (148). Therefore, MR antagonists (MRAs) therapy becomes crucial. Steroidal MRAs, such as spironolactone and eplerenone, are associated with significant side effects, particularly when combined with ACEIs or ARBs, often leading to hyperkalemia (149). Non-steroidal MRAs are considered more potent and have a lower risk of hyperkalemia compared to steroidal MRAs (150). Further analysis of data from the FIGARO-DKD trial, which assessed new-onset HF (hospitalization for HF in patients without a history of HF) and total HF hospitalizations, showed that finerenone significantly reduced the incidence of new-onset HF (1.9% vs. 2.8%; HR 0.68 [95% CI, 0.50–0.93]; P=0.0162). In the overall population, finerenone was associated with an 18% reduction in cardiovascular death or first hospitalization for HF, a 29% reduction in the risk of first hospitalization for HF, and a 30% reduction in total hospitalization for HF (151). The FIGARO-DKD trial findings indicated that finerenone reduced new-onset HF and improved HF outcomes in patients with CKD and T2DM, independent of a history of HF.Effective prevention of HF hospitalization by finerenone was also observed in the FIDELIO-DKD trial, which showed a 14% reduction in risk (152). A combined analysis of the FIDELIO-DKD and FIGARO-DKD trials found that the reduction in composite cardiovascular outcomes was primarily driven by a reduction in HF hospitalizations (HR 0.78; 95% CI 0.66–0.92) (153). A meta-analysis revealed that for most patients with T2DM and CKD, there were 10 additional cases of hyperkalemia per 1,000 patients over five years of non-steroidal MRA treatment, but a reduction of 16 deaths, 21 HF hospitalizations, and 14 cases of end-stage renal disease (154). This highlights the benefits of finerenone as a new-generation MRA, which outweigh its side effects.The ADA guidelines recommend adding MRAs if T2DM patients with hypertension are on three antihypertensive medications, including diuretics, and their blood pressure is not at target (117). Given that finerenone achieves equal concentrations in heart and kidney tissue—unlike the higher concentration of spironolactone and eplerenone in the kidney—and due to its higher selectivity and fewer side effects, it may be preferable to choose finerenone when adding an MRA for T2DM patients with HF or at high risk of HF (153, 155). The 2023 European Society of Cardiology (ESC) guidelines state that finerenone (Recommended for 1A) should be added to ACEIs or ARBs in patients with T2DM complicated by an eGFR > 60 mL/min/1.73 m² with a urinary albumin-to-creatinine ratio (uACR) ≥ 300 mg/g, or an eGFR of 25 to 60 mL/min/1.73 m² with a uACR ≥ 30 mg/g, to further reduce cardiovascular events and renal failure (156). However, research on whether to add RAAS agents to T2DM patients without hypertension or proteinuria, but at high risk of HF, is lacking.

### 7.3 Bariatric surgery

Metabolic surgery, which leads to long-term weight loss of 15-25%, has increasingly become a treatment for obesity and T2DM due to its positive impact on overall metabolism in these patients (157–159). Three large population studies have demonstrated a clear link between weight loss and the incidence of HF (160–162). In the SOS study, which included 2,003 patients who underwent bariatric surgery and 2,030 controls, the surgery cohort experienced the greatest weight loss (mean weight loss of 41 kg) and the lowest risk of HF at 1 year (160). Another study compared 25,804 patients who underwent gastric bypass surgery with 13,701 patients who underwent lifestyle changes, finding that the surgical cohort had a 46% reduction in the incidence of HF during a median follow-up of 4.1 years (161). Similarly, a large observational study in the United States of more than 180,000 insured individuals with obesity showed that after a median follow-up of 4 years, patients who underwent metabolic surgery had a 54% reduction in the risk of new-onset HF, with similar results observed in both patients with and without T2DM (162).A meta-analysis suggests that the cardioprotective effects of bariatric surgery are associated with the restoration of left ventricular hypertrophy and improvements in left ventricular geometry and diastolic function (163). Several studies have indicated that bariatric surgery may also have beneficial effects on subclinical myocardial dysfunction (164–168). The beneficial impact of metabolic surgery on the risk of HF in patients with T2DM may be attributed to both direct improvements in cardiac structure and function, as well as the reversal of hemodynamic changes and regenerative cell exhaustion. Additionally, metabolic surgery indirectly affects other risk factors such as hypertension, metabolic disturbances, and obesity (163, 169–172).

## 8 Best practices for clinicians

**Figure 2:**
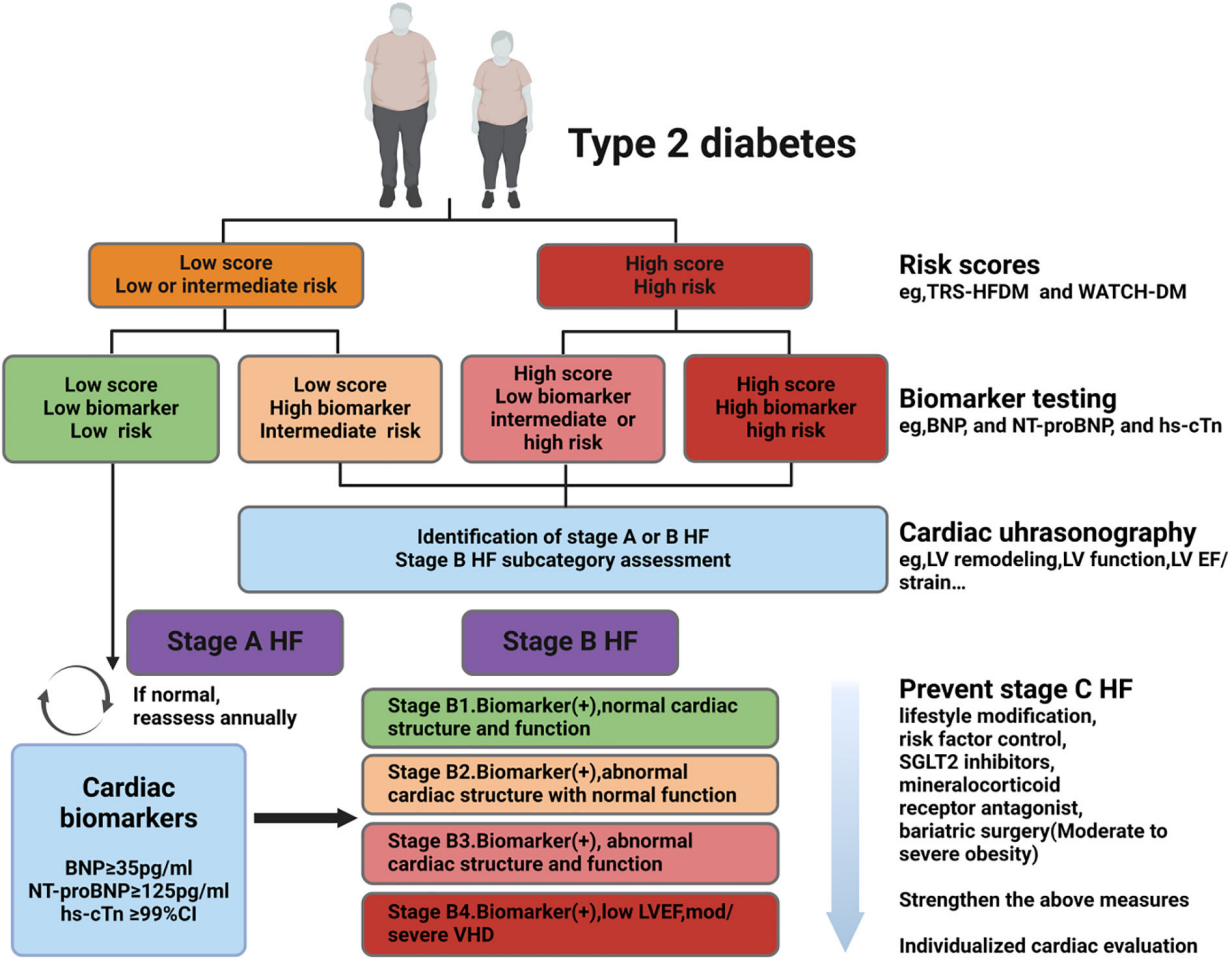
Early screening of HF in patients with T2DM. Screening strategy: i. Following ADA guidelines, it is recommended to screen all T2DM patients annually for Stage B HF using BNP (≥50 pg/mL) or NT-proBNP (≥125 pg/mL); clinical risk scores can be used as an initial screening tool, with high-risk patients directly undergoing echocardiography. ii. Subclassification of B1-B4 stages: Based on biomarker abnormalities and echocardiographic results, Stage B is further subdivided, necessitating intensified lifestyle interventions, weight loss, and early initiation of SGLT2 inhibitors. iii. In patients with comorbid CKD (uACR ≥30 mg/g or reduced eGFR): Immediately initiate combination therapy with SGLT2 inhibitors and finerenone. iv. Treatment intensity: Gradually escalate intervention measures with the progression of Stage B. T2DM:type 2 diabetes mellitus, HF: heart failure, hs-cTn: highly sensitive-cardiac troponin, BNP: N-terminal pro B-type natriuretic peptide, NT-proBNP: N-terminal pro B-type natriuretic peptide, LV: left ventricle, EF: ejection fraction, SGLT2: sodium glucose cotransporter 2,ADA: American Diabetes Association, CKD: chronic kidney disease, VHD: valvular heart disease. Created by BioRender.com.

Based on current understanding of early screening for HF in T2DM, the following process is summarized (**Figure 2**): First, regular screening is recommended.The ADA guidelines suggest annual screening (84). Second, a clinical risk score can be useful as an initial step in screening. For cardiac biomarker screening, although it is reasonable to conduct it annually as recommended by the ADA, the clinical validity and cost-effectiveness of this approach for the entire T2DM population require further evaluation. Echocardiography may also be considered directly, without prior cardiac biomarker assessment, in patients with high clinical risk scores. This is because the presence of cardiac structural and functional abnormalities in these individuals can identify stage B HF, even in the absence of biomarker testing, necessitating aggressive intervention (7, 26). Third, elevated clinical risk or abnormal levels of cardiac biomarkers (e.g., BNP ≥50 pg/mL, NT-proBNP ≥125 pg/mL, or hs-cTn ≥99th percentile), in combination with echocardiogram evaluation, further subdivides stage B HF (B1-B4). This should prompt aggressive risk factor modification, lifestyle interventions, increased physical activity, weight loss, and early initiation of SGLT2 inhibitors, which should be progressively intensified as the stage B classification advances (7). Fourth, if comorbid CKD is present (uACR ≥30 mg/g or reduced eGFR), a combination of SGLT2 inhibitors and finerenone should be initiated immediately. Finally, patients at high risk should seek evaluation from a cardiologist at or after starting the recommended preventive measures, especially those with stage B3 or B4 HF.

## 9 Current limitations and future directions

Over the past decade, advancements in the field of HF in T2DM have primarily focused on the treatment of symptomatic HF, with limited evidence supporting the assessment and prevention of stage B HF progression to stage C HF. Specifically, tools for early risk stratification and the application of biomarkers have lacked large-scale validation, limiting the formation of clinical consensus. Existing predictive models primarily rely on clinical variables (e.g., age, gender, medical history), without fully incorporating subclinical cardiac structural changes or functional abnormalities (e.g., myocardial strain abnormalities).Additionally, the development of these models is based on regional and ethnic heterogeneity, which constrains their accuracy and generalizability. Large-scale cohort studies stratified by age, sex, and ethnicity are warranted in T2DM populations. By integrating clinical variables, novel biomarkers, and high-sensitivity indicators of cardiac dysfunction, these studies will enable the development of efficient predictive models. The discovery, clinical validation, and application of effective biomarkers are essential. Notably, as a validated surrogate marker of insulin resistance, the triglyceride-glucose (TyG) index significantly correlates with early subclinical HFpEF risk in T2DM and predicts HFpEF prognosis (173–175). This biomarker offers a novel strategy for identifying stage B HF and stratifying risk in T2DM. Emerging proteomic, metabolomic, and epigenetic technologies reveal promising biomarkers including sST2, galectin-3, and non-coding RNAs, which correlate with early myocardial fibrosis or metabolic dysregulation (176–178). However, their prognostic utility for HF progression in T2DM requires validation.Novel ultrasound techniques such as speckle-tracking echocardiography enhance diagnostic accuracy for stage B HF by detecting subtle myocardial strain abnormalities. Continued innovation in cardiac ultrasound will undoubtedly refine HF risk assessment. Moreover, recent research has achieved substantial progress in elucidating mechanisms underlying diabetes-induced myocardial damage, with key insights into mitochondrial dysfunction, apoptosis and ferroptosis regulation (179, 180). Developing precisely targeted therapeutic agents against these mechanisms holds promise for early and precise prevention of T2DM-mediated HF progression.

## 10 Conclusion

As a major cardiovascular complication in T2DM, HF poses significant clinical challenges.Advances in clinical risk stratification, biomarker profiling, and echocardiographic techniques for predicting HF onset, coupled with the development of novel therapeutics such as SGLT2 inhibitors, MRAs, and GLP-1 receptor agonists, hold promise for reducing the contemporary burden of HF in T2DM. Nevertheless, effective tools for screening high-risk individuals and targeted interventions to mitigate T2DM-driven pathophysiological mechanisms of HF have yet to be established.Consequently, bridging these translational gaps demands sustained advancement across developing accurate HF risk-stratification tools, identifying robust biomarkers and next-generation echocardiographic innovations, creating molecularly targeted therapeutics and ensuring their effective clinical translation.
